# Methods, development and applications of small-angle X-ray scattering to characterize biological macromolecules in solution

**DOI:** 10.1016/j.crstbi.2020.08.004

**Published:** 2020-08-27

**Authors:** Stefano Da Vela, Dmitri I. Svergun

**Affiliations:** EMBL Hamburg Unit, c/o DESY, Notkestr. 85, 22607, Hamburg, Germany

**Keywords:** SAXS, Structural biology, Proteins, Complexes, Biophysics

## Abstract

Applications of small-angle X-ray scattering (SAXS) in structural biology are reviewed. A brief introduction of the SAXS basics is followed by the presentation of the structural features of biological macromolecules in solution that can be assessed by SAXS. The approaches are considered allowing one to obtain low resolution three-dimensional (3D) structural models and to describe assembly states and conformations. Metrics and descriptors required for the assessment of model quality are presented and recent biological applications of SAXS are shown.

## Highlights

• Small-angle X-ray scattering (SAXS) is a powerful technique in structural biology.
• SAXS allows to study biological macromolecules in close to native solutions.
• Low-resolution models of proteins, nucleic acids and complexes can be obtained.
• Atomic models can be integrated using hybrid analysis approach.
• SAXS is applicable to partially or completely disordered macromolecular states.

## Graphical abstract

**Figure:**
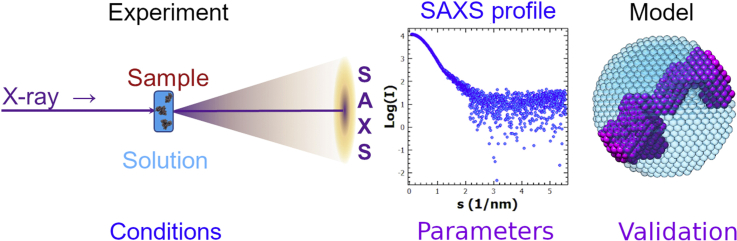

Small-angle X-ray scattering (SAXS) is an established method for structural characterization of samples at resolutions between 1 nm and 1000 nm. SAXS is sensitive to both ordered and not-ordered features in the sample and it does not require crystallization, fixation, or vitrification procedures. In a SAXS experiment (Fig. 1A), a collimated, monochromatic X-ray beam hits the sample and the radiation scattered at low angles (typically a few degrees) is recorded by a detector. In the present review, applications to dilute solutions of biological macromolecules are considered. The scattering originating largely from the electrons in the sample is typically isotropic and a radial averaging of the 2D scattering pattern yields a 1D intensity curve (Svergun et al. 2013). The signal comes not only from macromolecules themselves, also from the buffer and the surrounding container: these unwanted contributions are removed by background subtraction. The data quality depends crucially on the accuracy of this operation (the buffer must be “matching” the one surrounding the macromolecules). The subtracted SAXS profile yields the intensity from the macromolecules as a function of the scattering angle (Fig. 1B,C,E). The scattered intensity is a faithful reflection of the structure of the macromolecule of interest for dilute solutions, if contributions from aggregates, self-association or positional correlations between different molecules are neglectable. On-line size-exclusion chromatography (SEC-SAXS) (David & Pérez 2009), which became popular in recent years, can be employed to separate the macromolecule or complex of interest from aggregates, higher oligomers or other interfering components (Fig. 1D,F), provided the thermodynamics and kinetics of formation of the interfering species allow for such a separation. A particularly challenging application of SAXS, facilitated by SEC-SAXS, is the study of solubilized membrane proteins, see e.g. (Molodenskiy et al. 2020) and references therein. The physics of scattering is described in detail in (Svergun et al. 2013): here we summarize a few fundamentals for a better understanding. The scattering intensity I is expressed as a function of momentum transfer, *s*, with the dimensionality of (length)^−1^ (see Fig. 1B,E; note that various symbols are used in the literature, most common being s, q and Q). I(s) is proportional to the square of the Fourier transform of the excess electron density of the particle compared to the surrounding buffer. The structural features of the macromolecule in real space (after ensemble and orientation average) are thus encoded into the scattering intensity in the Fourier (reciprocal) space. For dilute samples, the scattering is usually proportional to the averaged scattering from a single particle. At higher concentrations, modulations due to intermolecular correlations can be observed (so-called structure factor (Svergun et al. 2013)); a concentration series is often measured to extrapolate the data to “infinite dilution”. As evident from equation (Fig. 1E(4)) the scattering intensity is proportional to the squared volume of the macromolecule; the SAXS signal is thus more sensitive to higher molecular mass species.

**Fig. 1:**
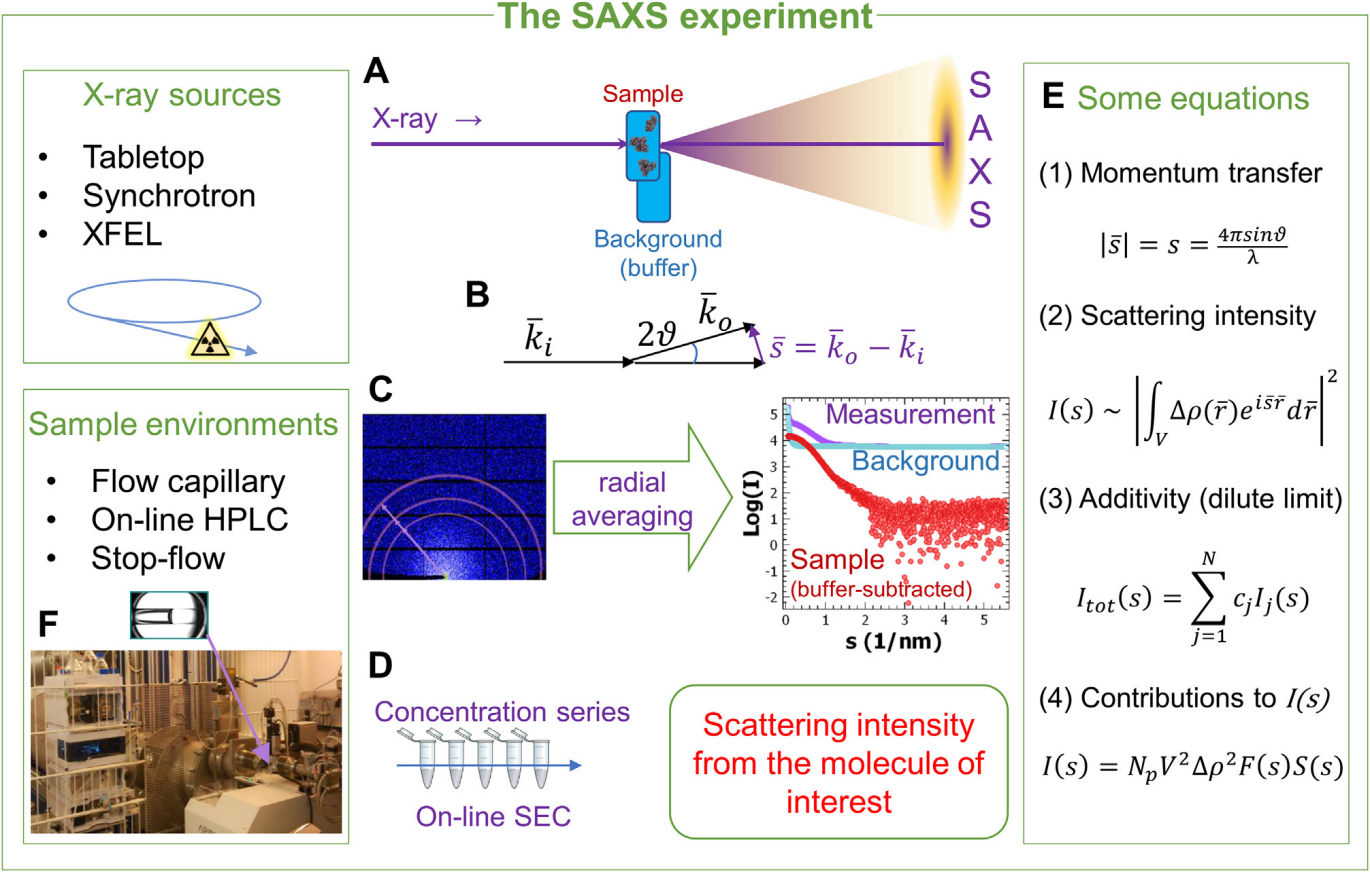
**A**. A biological SAXS experiment: an X-ray beam impinges on the sample, usually an aqueous solution of macromolecules, and the X-rays scattered at low angles are collected by a detector. Each sample measurement is paired with an appropriate background measurement. **B**. Geometry of the SAXS experiment. The incoming radiation is represented by the wave-vector $\bar{k_{i}}$ , and the radiation (elastically) scattered at an angle 2θby the wave-vector $\bar{k_{o}}$, of identical magnitude. The scattered intensity is recorded as a function of the modulus of the scattering vector $\bar{s}={\bar{k}}_{0}-\bar{k_{i}}$. **C**. The radiation collected by the detector is radially averaged to obtain the SAXS profiles of sample and background, and their difference yields the scattering from the macromolecules. **D**. Concentration-dependent effects are usually accounted for by performing the measurement on a concentration series; on-line size exclusion chromatography (SEC) may be applied to remove aggregates. **E**. Equations fundamental for biological SAXS. (1) definition of the momentum transfer s as used in this review (modulus of the scattering vector $\bar{s}$ : see Fig.1B), λ is the X-ray wavelength; (2) for dilute samples, the scattered intensity I(s) is proportional to the square of the Fourier transform of the electron density distribution ($\Delta \rho \left(\bar{r}\right)$) of the macromolecule with respect to the buffer. (3) For dilute systems, the scattering is additive, and the total scattering intensity $I_{tot}\left(s\right)$ of a mixture is the linear combination of the scattering intensities $I_{j}\left(s\right)$of its N components, weighted by their volume fractions, $c_{j}$. (4) The scattering intensity is related to the number of macromolecules per unit volume ($N_{p}$), the square of their volume V and of their electron density contrast Δρ, the scattering due to the shape of the particle F(s) and a interference term S(s) known as structure factor, related to the relative positions of the macromolecules; S(s) approaches 1 for infinite dilution. **F**. Typical sample environments. Inset: the sample environment of the EMBL beamline P12 (Petra III storage ring, DESY, Hamburg).

The SAXS data are measured between a minimum (${\text{s}}_{\text{min}}$) and a maximum (${\text{s}}_{\text{max}}$) values of the momentum transfer (Fig. 2A). Due to the reciprocal relation between the scattering profile and real space length scales, this translates the nominal resolution covered by SAXS from about $2\pi /{\text{s}}_{\text{min}}$ at large length scales to $2\pi /{\text{s}}_{\text{max}}$ at small length scales. Particle size, molecular weight, volume (Fig. 2A and B), overall compactness and anisometry (Fig. 2C,E) are obtained model-free from the data (see also (Svergun et al. 2013) and the classical papers referenced therein). The radius of gyration, $\left({\text{R}}_{\text{g}}\right)$ is the weighted root mean square of the intramolecular distances with respect to the centroid of the electron density, representing an effective size for the macromolecule. ${\text{R}}_{\text{g}}$ is routinely obtained from the slope of the low-angle “Guinier” region, which for monodisperse dilute non-interacting solutions is linear in a plot of ln(I) *vs.* $s^{2}$ as $\ln\left(I\right)=\ln\left(I\left(0\right)\right)-\frac{1}{3}R_{g}^{2}s^{2}$. I(0) is proportional to the squared number of excess electrons in the macromolecule compared to the buffer, and if the solute concentration is available I(0) allows one to assess the molecular mass. Concentration-independent estimates of the molecular mass can be obtained from the SAXS curves using different approaches (Fischer et al. 2010) (Rambo & Tainer 2013) (Hajizadeh et al. 2018). SAXS curves usually rapidly decay with the angle and the scattering at higher angles is mainly due to the macromolecule–solvent interface (“Porod region”). The excluded volume of the hydrated macromolecule (Vp) can be extracted using the “Porod invariant” using a weighted integral of the intensity (Svergun et al. 2013). A Fourier transformation of the intensity, the pair distance distribution function P(r), yields a real-space representation of the intra-molecular distances (Fig. 2E). P(r) equals to zero if *r* exceeds the maximum intramolecular distance ($D_{max}$), and this allows one to estimate $D_{max}$ from the experimental data using the so-called indirect transform methods. The shape of P(r) further provides indications on the overall particle shape (Svergun et al. 2013). Machine-learning methods are becoming increasingly popular for SAXS data analysis; a recent approach to classify the overall shape from SAXS data is implemented in the program DATCLASS (Franke et al. 2018).

**Fig. 2:**
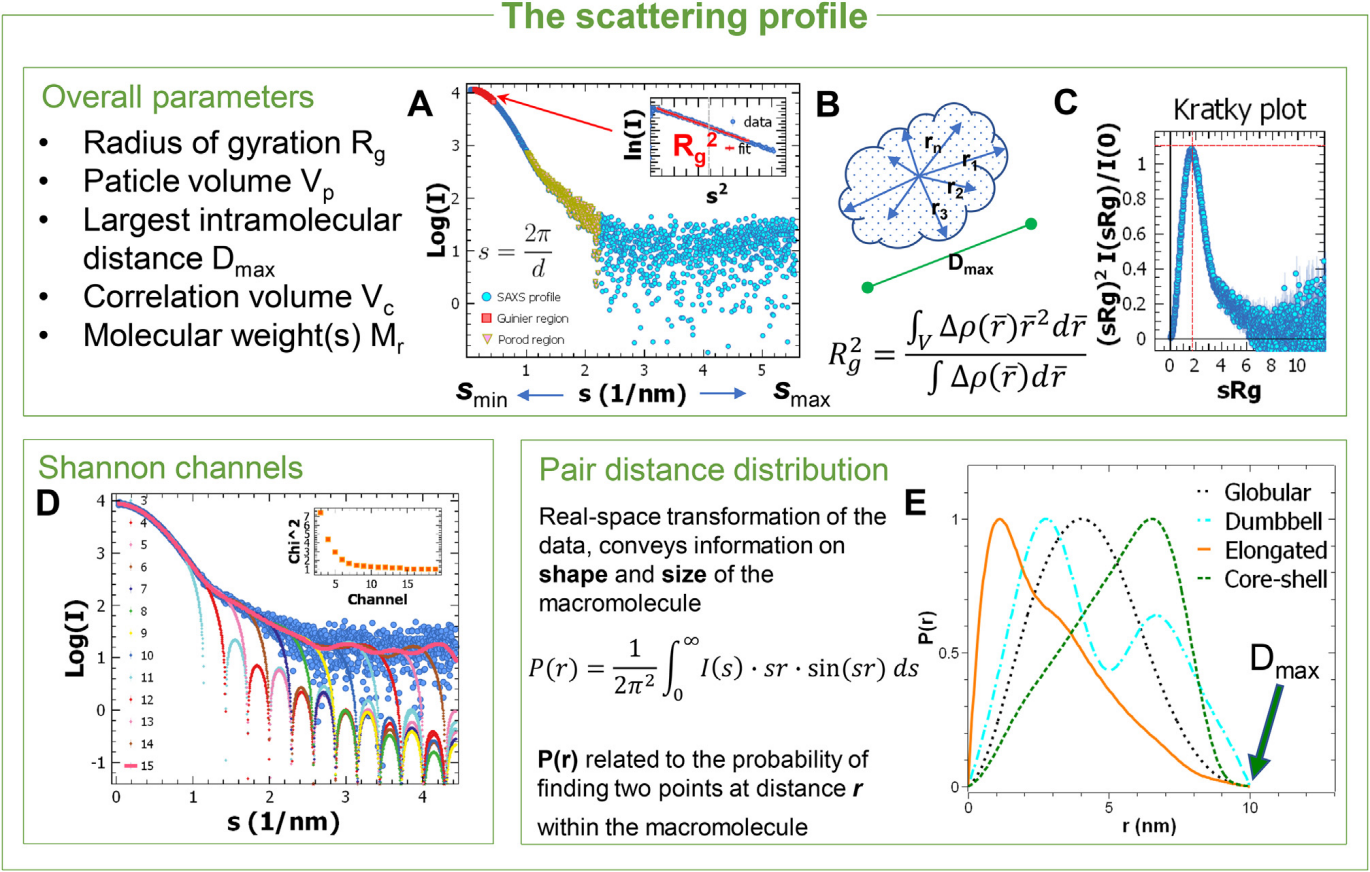
**A**. A SAXS profile (in semi-logarithmic scale) with highlighted Guinier and Porod regions. The value of the momentum transfer is reciprocally related to the size (d) in real space. Inset: linear region of the Guinier plot showing the extraction of the radius of gyration $R_{g}$ from the SAXS profile. Several methods are available to assess the molecular mass (see main text). **B**. Illustration of the largest intramolecular distance, $D_{max}$ and of the meaning of the $R_{g}$ of a macromolecule of arbitrary shape. **C**. Typical dimensionless Kratky plot for a compact/globular macromolecule. Deviations from the bell-shaped plot with maximum at (1.732,1.104) -red dashed cross-, point to strong anisometry or structural disorder. **D**. The Shannon channels formalism helps selecting the s-range of experimental data useful for modelling, that is containing the information needed for structural reconstruction while avoiding the inclusion of potentially misleading high-angle contributions. Shown is an experimental SAXS profile, which can be reconstructed (solid line) based on the first 15 Shannon channels (dotted curves). The inset shows the selection of the optimal number of channels to have good agreement of the reconstructed curve with the data. **E**. The scattering intensity can be transformed into the pair distance distribution function (PDDF or P(r), with r a real-space distance); the maximum size $D_{max}$is the r value at which P(r) returns to 0. The appearance of P(r)provides an intuitive feeling of the shape class of the macromolecule, as well as an independent way to obtain $R_{g}$.

The SAXS profile of a macromolecule can be described by a finite set of values spaced at $\pi /{\text{D}}_{\text{max}}$ (Shannon channels). An experimental profile can be reconstructed increasingly well by truncated Shannon approximation employing an increasing number of these values, up to an optimum after which the reconstruction overfits the data. Determination of the optimum number of Shannon channels (program SHANUM (Konarev & Svergun 2015)) excludes high-angle noisy contributions which do not contain useful information (Fig. 2D).

A useful presentation of SAXS data is the Kratky plot of $I\left(s\right)s^{2}\;vs\;s$ and its dimensionless version (Durand et al. 2010) (Fig. 2C). Compact, globular molecules display a bell-shaped plot, partially folded or elongated molecules show a maximum shifted to higher ${\text{sR}}_{\text{g}}$values, whereas unfolded, disordered molecules feature a monotonic increase at higher angles. The volume of correlation ($V_{C}$) calculated from the SAXS profile is a parameter that, other than providing an estimate of the molecular mass (Rambo & Tainer 2013), can be used to assess the folding state of proteins (Watson & Curtis 2014), as for compact proteins $V_{C}$ exhibits approximately a power-law scaling with the number of amino acids.

SAXS is used for structural modelling of macromolecules, oligomers, complexes, and large assemblies but also for assessing structural responses to changes in the solution conditions, including the addition of ligands. High-resolution models from crystallography, electron microscopy or NMR can be utilized and assembled into larger complexes to be further tested and selected against the SAXS data. The modelling accuracy can be improved by additional information from other experiments, such as crosslinking, FRET or H/D exchange. The modelling approaches can be divided into *ab initio* methods (Fig. 3A) and those employing existing structures or fragments (Fig. 3B). Popular *ab initio* approaches (Fig. 3A) use a coarse-grained description of the macromolecule. In a uniform approximation, 3D particle shape can be modelled by close-packed beads (“dummy atoms”). The modelling starts by randomly assigning the beads to either the particle or solvent. The assignment is randomly changed following an iterative simulated annealing algorithm, which optimizes a score function f(X) including the fit quality to the data and a set of penalties ensuring feasibility (e.g. interconnectivity) of the model. For proteins one may employ “dummy residues” for a similar procedure, introducing the *a priori* knowledge that proteins are folded linear polymers of amino acids. Implementations of the two approaches are found e.g. in programs DAMMIF and GASBOR from the ATSAS suite (Franke et al. 2017). Oligomeric equilibria can be treated *ab initio* as well exploiting the additivity of scattering (Fig. 1E(3)). Recently, an alternative *ab initio* approach was proposed (Grant 2018) utilizing an average of multiple 3D density maps, each iteratively reconstructed from the scattering intensity within a spherical volume of radius D_max_/2.

**Fig. 3:**
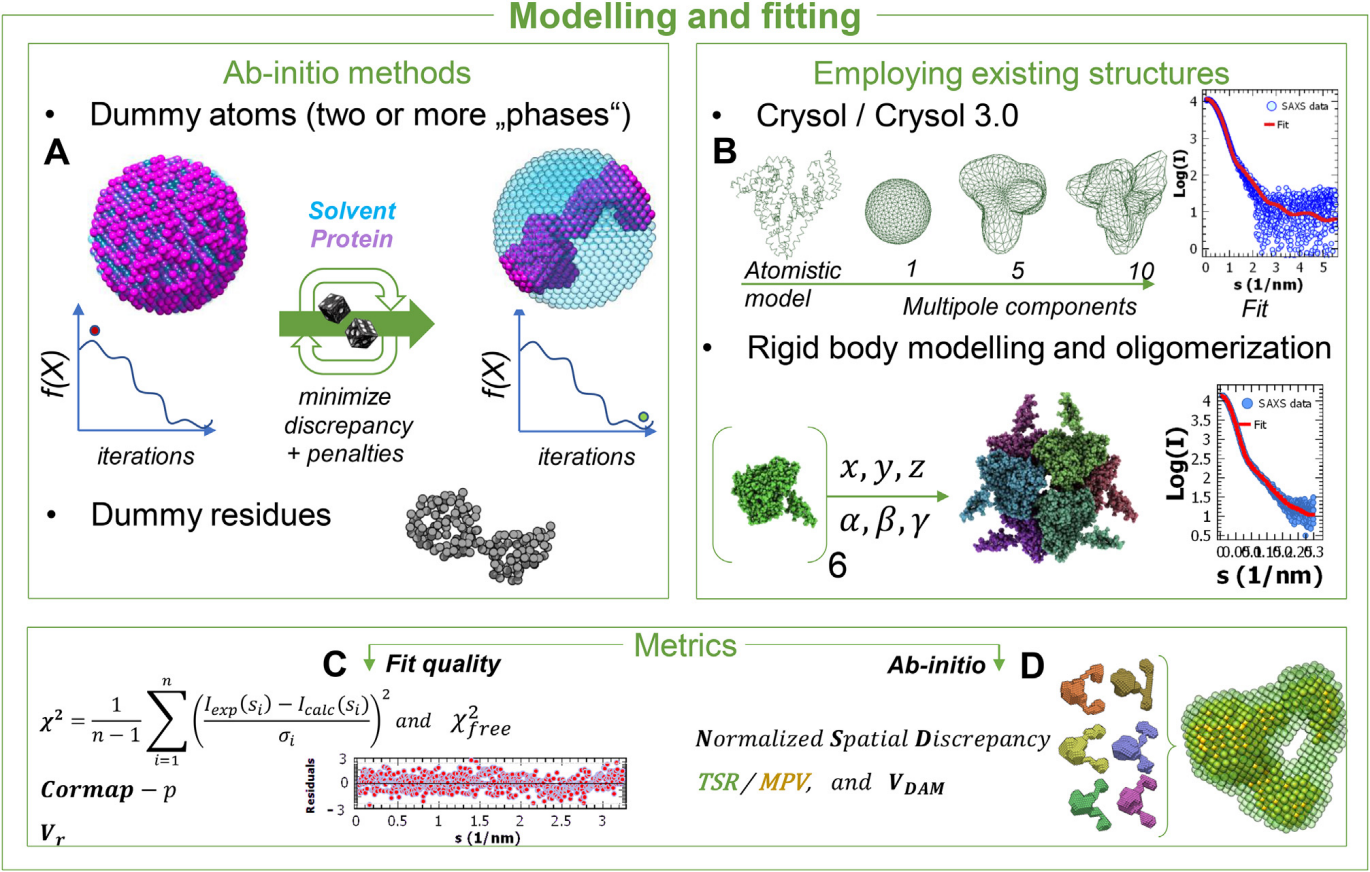
**A.** Illustration of finite elements *ab initio* modelling procedures to obtain SAXS models. The macromolecule is coarse-grained as an ensemble of packed beads (dummy atoms) or as a chain of dummy residues. An iterative simulated annealing algorithm changes the configuration of the beads or residues minimizing the score function f(X), which includes the discrepancy to the SAXS data as well as structural constraints to ensure a physically plausible solution. **B**. Example of comparison of atomistic structures with SAXS data. Atomistic models can be approximated by a series of spherical harmonics, for an efficient calculation of the scattering intensity. Rigid-body approaches allow to model oligomers and complexes (the example is a hexamer of E.coli GadA (sasbdb accession code: SASDB33) **C**. Some metrics for the assessment of fit quality. Inset: plot of residuals as a function of s. The equation for the reduced χ^2^ is shown: n is the number of points in the SAXS curve, σ_i_ the error associated to the i-th point and I_exp_ (s_i_) and I_calc_ (s_i_) are the intensity values at the i-th point for the experimental and fitted curve, respectively. **D**. Metrics applied to *ab initio* reconstructions. The inset shows six dummy atom models, fitting the same curve but displaying variability. On the right, superposition of the most populated volume (MPV, yellow beads) with the total spread region (TSR, translucent green beads). The TSR and MPV were calculated using DAMAVER (Volkov & Svergun 2003).

When high-resolution models are available from other methods, their calculated scattering profiles can be compared to the SAXS data (Fig. 3B). A rapid calculation algorithm employs a multipole expansion in a series of spherical harmonics (program CRYSOL (Svergun et al. 1995)). The multipole representation provides a mean for rapid computation of translations and rotations in real space (Svergun et al. 2013). This approach allows for rigid body modelling of complexes, oligomers, and even oligomeric mixtures, the latter represented by linear combinations of scattering intensities from their components (programs SASREF/SASREFMX) (Franke et al. 2017). Alternative programs for the use of high-resolution models in SAXS analysis are e.g. FoXS, FoXSDock and MultiFoXS (Schneidman-Duhovny et al. 2016).

Several metrics apply to SAXS-based structural models (Fig. 3C and D). The fit quality is generally assessed by the $\chi^{2}$ discrepancy parameter, Fig. 3C ($\chi^{2}$ ~1 for good fits). Its value depends on correctly estimated experimental errors and could be artificially lowered by too large error bars in the intensities. A plot of the error-normalized fit residuals helps one in identifying regions in which the data depart from the fitted curve. A $\chi_{free}^{2}$ was proposed as a metric more robust against overfitting, calculating the goodness-of-fit of the model against the downsampled SAXS profile (Rambo & Tainer 2013). Additionally, a volatility of ratio ($V_{r}$) (Hura et al. 2013) can be employed for assessing the fit quality and comparing SAXS profiles. V_r_ may be useful to detect subtle structural changes and to produce similarity maps for pairwise comparisons in parametric studies. The CorMap test (Franke et al. 2015) allows for quantitative comparison of SAXS profiles without the knowledge about the associated errors. It utilizes only the residuals between the intensities and yields the probability that the two data sets are statistically similar.

The reconstruction of a 3D scattering object from 1D data does not have a unique solution leading to variability of *ab initio* reconstructions, and comparison of the results of repeated reconstructions is often useful. Metrics such as the normalized spatial discrepancy (NSD) are applied to the set of solutions, yielding a stability measure (average NSD significantly exceeding unity indicates ambiguous solutions). For dummy-atom modelling, inspection of the total spread region (TSR) and of the most populated volume (MPV) reveal the regions of increased variability and also features common to the different models (Volkov & Svergun 2003). Each dummy-atom model has an associated volume ($V_{DAM}$), which provides another estimate of the volume (and thus the molecular mass) of the macromolecule.

Different approaches exist for SAXS-assisted modelling of macromolecules featuring flexibility and disorder (Fig. 4). Conformational heterogeneity can be reflected in the models to a varying extent, from disordered regions represented by their “average” conformation, to ensembles spanning the same conformational space as the molecule of interest. Prior information usually helps in the selection of the appropriate type of modelling, but also the intensity decay in the Porod region can be useful (as a rule of thumb, slower decays point to increased flexibility). Validation schemes to detect the most relevant conformational features of non-unique or ensemble reconstructions have been suggested, based on molecular dynamics (Wright et al. 2020) or target-decoy methods borrowed from ligand docking (Luo et al. 2014).

**Fig. 4:**
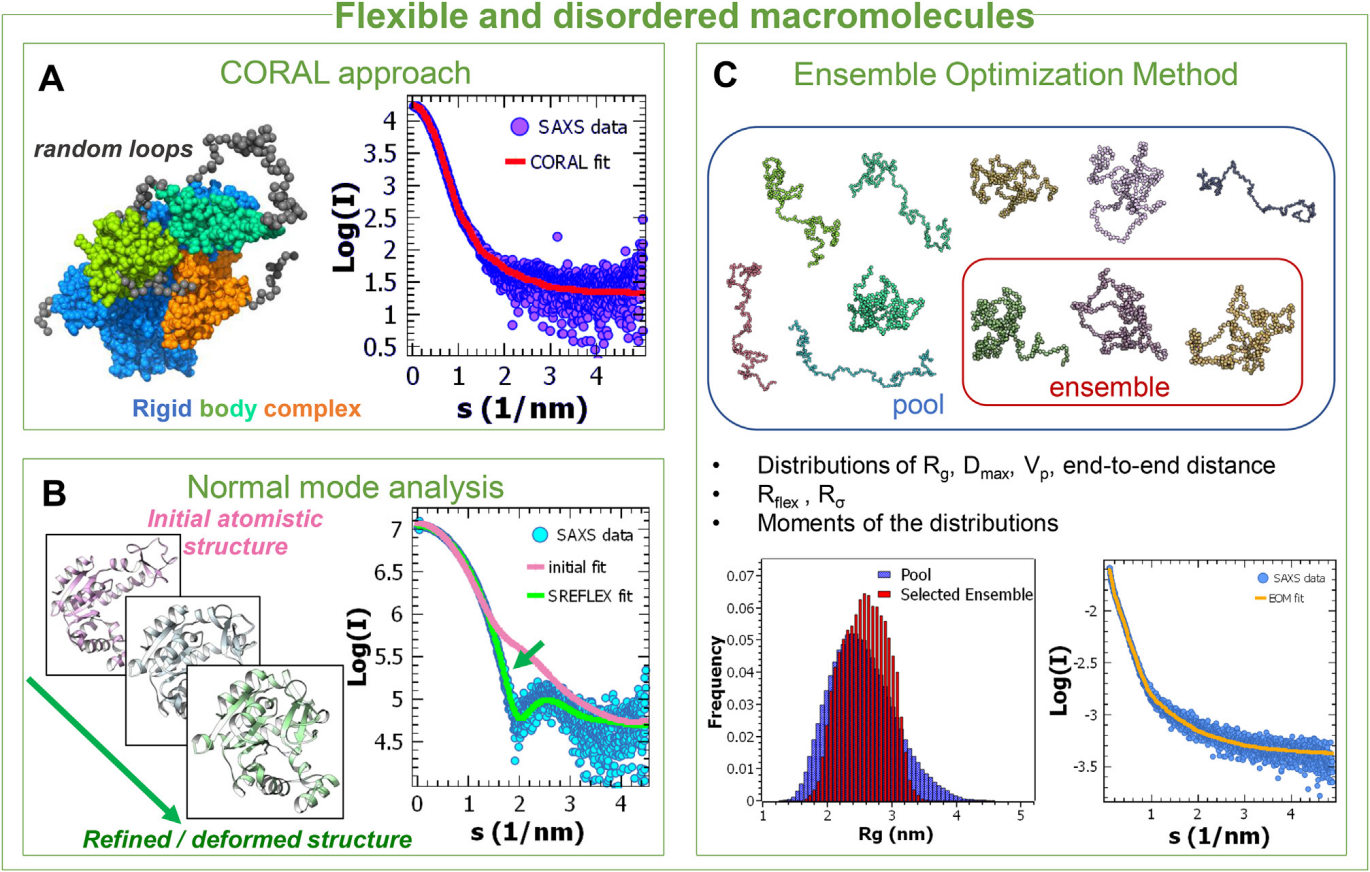
**A**. CORAL approach for modelling un-structured loops and termini when incomplete high-resolution structures are available. The latter are used in rigid body modelling, while the random parts are optimized against the data in the form of self-avoiding peptide-like chains. (SASBDB accession code: SADDR9). **B**. Illustration of normal mode analysis-based fit, as implemented in SREFLEX. An initial high-resolution structure is deformed to improve its fit to the data. **C**. Illustration of the Ensemble Optimization Method (EOM), selecting an ensemble of conformations from a random pool and yielding distributions of size parameters for the flexible molecule.

If part of the structure is known but the macromolecule features portions not seen by high resolution methods like crystallography, a hybrid of rigid-body modelling and optimization of flexible loops and termini (program CORAL (Petoukhov et al. 2012), Fig. 4A) can be used to fit the SAXS data and yield structures depicting average conformations of the flexible parts. A possible conformational heterogeneity of these parts is reflected by increasing variability of the reconstructions.

The macromolecular motions can be considered explicitly in methods employing normal mode analysis (NMA), such as SREFLEX (Panjkovich and Svergun, 2015) (Fig. 4B). If an atomistic structure of the molecule is available, these methods generate conformationally altered structures, based on coarse-grained dynamics of the macromolecule. These models are then ranked based on their fits to the SAXS data.

Intrinsically disordered proteins, or those containing significant unfolded fragments, are best modelled considering co-existing ensembles of conformers. The first approach developed to this end, the ensemble optimization method (EOM) (Bernadó et al. 2007), selects structures from a pool of models with varying random chains against the data employing a genetic algorithm, to yield a mixture (ensemble) whose average scattering fits the data (Fig. 4C). EOM provides the distributions of dimensional parameters in the selected ensemble ($R_{g}$, $D_{max}$) to assess the flexibility; additionally, quantitative metrics describing the conformational disorder are computed to characterize the degree of disorder of the flexible regions. Other ensemble-based methods are presently available, e.g. Ensemble-Refinement of SAXS (EROS (Różycki et al. 2011)), which employs coarse-grained simulations to compute the pool of models.

Examples of recent applications of biological SAXS utilizing various analysis approaches are given in Fig. 5. In the study of a toxin-antitoxin complex MbcT-MbcA from *Mycobacterium tuberculosis* (Fig. 5A), the *ab initio* modelling well reproduces the overall toroidal shape of the complex and the SAXS data can also be neatly fitted by the scattering computed from the crystal structure (Freire et al. 2019). Fig. 5B illustrates a validation in solution of the heterodimeric complex between the tRNA methyltransferase Trm7 and its partner subunit Trm734 from *Saccharomyces cerevisiae* (Hirata et al. 2019). Here, the SAXS data are used to model the full structure, including two disordered C-terminal regions of Trm7, not visible in the crystallographic electron density map. An application of NMA to study flexible proteins (Manalastas-Cantos et al. 2019) is presented in Fig. 5C. Here, the crystal structure of the condensin HEAT-repeat subunit Ycg1 needs to be deformed to fit the SAXS data, highlighting the different conformation of the protein in solution, and flexibility is further observed in the modelling of the Ycg1-Brn1 complex. Finally, Fig. 5D illustrates the application of SAXS to study pharmaceutical formulations (Xu et al. 2019). The structure factor of concentrated antibody solutions (see Fig. 1E (4)) was obtained in the presence of alanine as a co-solute. SAXS, yielding a simultaneous view of macromolecular conformations and unspecific interactions, allows the optimization of colloidal and conformational stability at different solution conditions. Overall, SAXS is becoming popular in pharmaceutically relevant applications, e.g. for high-throughput characterization of binding of small molecule ligands to proteins (Chen et al. 2020).

**Fig. 5:**
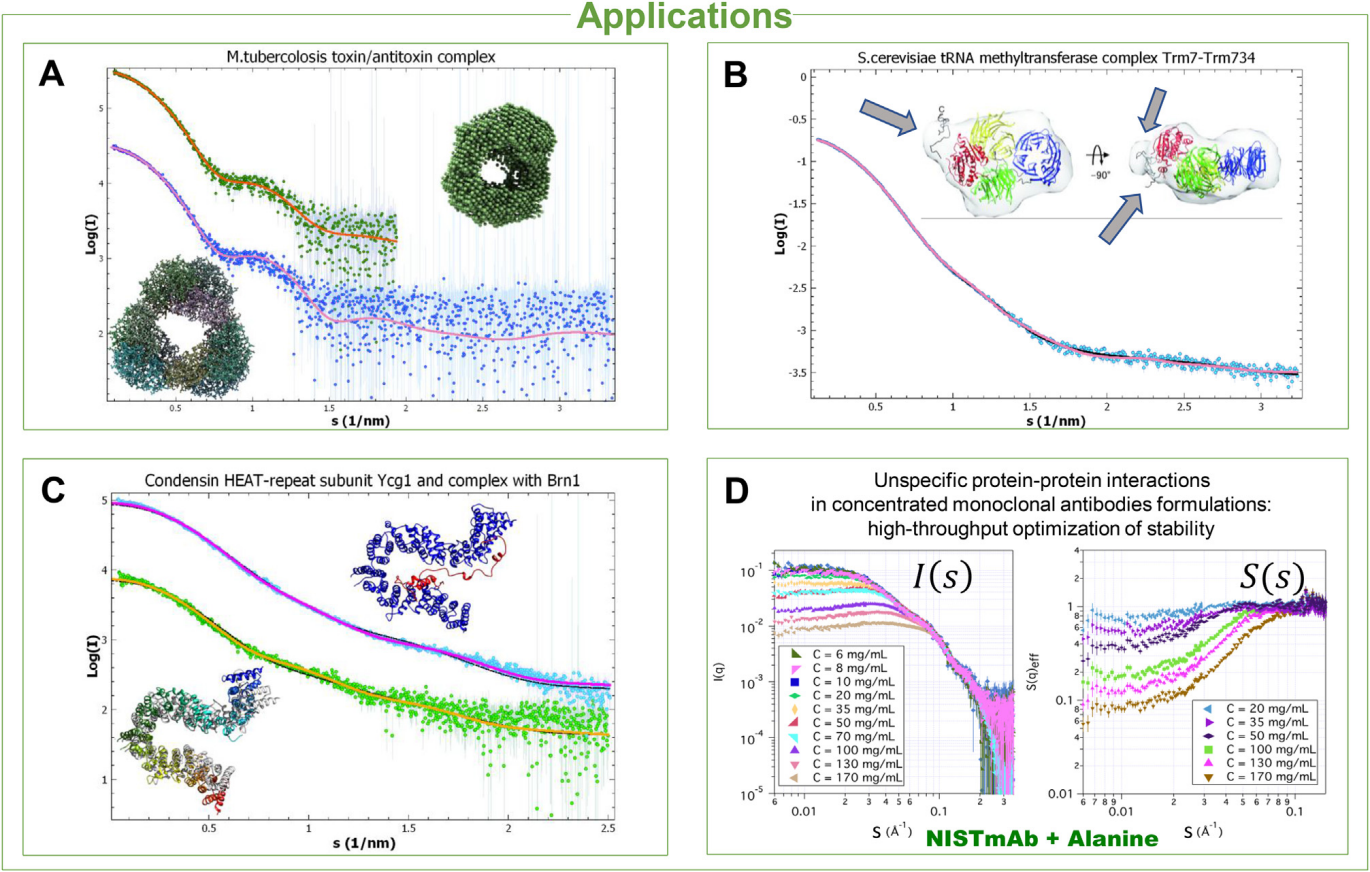
**A**. Study of a toxin-antitoxin complex from *Mycobacterium tuberculosis*, MbcT-MbcA (SASBDB accession code: SASDD33). Upper curve (vertically displaced for clarity) and model: fit and outcome of *ab initio* modelling against a restricted angular range of the SAXS data. Lower curve and model: fit of the high-resolution crystal structure of the complex, validating the heterododecameric assembly in solution. **B**. Study of a tRNA mehtyltransferase complex from *Saccharomyces cerevisiae*, Trm7-Trm734 (SASDDR3). Inset: CORAL model overlaid onto an *ab initio* model from the same data, in two orientations (reproduced from (Hirata et al. 2019), with permission). The gray arrows point to the modelled C-terminal disordered loops. The fit for the CORAL model is the light pink continuous curve, the thin dark line is the fit of the *ab initio* model.**C**. Application of NMA to model the condensin HEAT-repeat subunit Ycg1 and the Ycg1-Brn1 complex (SASDFC4, SASDFD4). Lower curve and models: fit of the NMA-based structure to the SAXS data. The fitted structure is shown as ribbon, in rainbow color scheme, superimposed to the original crystal structure (in grey). Upper curve (shifted vertically for clarity), and model: result of Ycg1–Brn1 complex SAXS profile fitting and modelling with NMA. The thin dark lines are the initial fits from the crystal structures. **D**. Scattering intensities and structure factors from the study of structure and stability of antibodies in concentrated formulations. The values of S(s)<1 at high concentrations in the presence of alanine indicate overall repulsive effective protein–protein interaction in these concentrated solutions (adapted from (Xu et al. 2019), with permission).

Summarizing, SAXS is a versatile tool in structural biology, offering broad possibilities for sample characterization in solution at the nm scale. Thanks to straightforward integration with other techniques, SAXS plays an increasing role in the validation and study of conformations and assemblies of biological macromolecules in solution.

## CRediT authorship contribution statement

**Stefano Da Vela:** Writing - original draft, Visualization. **Dmitri I. Svergun:** Conceptualization, Writing - review & editing, Supervision, Funding acquisition.

## Declaration of Competing Interest

The authors declare that they have no known competing financial interests or personal relationships that could have appeared to influence the work reported in this paper.

## References

[bib1] Bernadó P., Mylonas E., Petoukhov M.V., Blackledge M., Svergun D.I. (2007). Structural characterization of flexible proteins using small-angle X-ray scattering. J. Am. Chem. Soc..

[bib2] Chen P.-c., Masiewicz P., Perezb K., Hennig J. (2020). Structure-based screening of binding affinities via small-angle X-ray scattering. IUCrJVolume.

[bib3] David G., Pérez J. (2009). Combined sampler robot and high-performance liquid chromatography: a fully automated system for biological small-angle X-ray scattering experiments at the Synchrotron SOLEIL SWING beamline. J. Appl. Crystallogr..

[bib4] Durand D., Vivès C., Cannella D., Pérez J., Pebay-Peyroula E., Vachette P., Fieschi F. (2010). NADPH oxidase activator p67phox behaves in solution as a multidomain protein with semi-flexible linkers. J. Struct. Biol..

[bib5] Fischer H., de Oliveira Neto M., Napolitano H.B., Polikarpov I., Craievich A.F. (2010). Determination of the molecular weight of proteins in solution from a single small-angle X-ray scattering measurement on a relative scale. J. Appl. Crystallogr..

[bib6] Franke D., Jeffries C.M., Svergun D.I. (2015). Correlation Map, a goodness-of-fit test for one-dimensional X-ray scattering spectra. Nat. Methods.

[bib7] Franke D., Petoukhov M., Konarev P., Panjkovich A., Tuukkanen A., Mertens H., Kikhney A., Hajizadeh N.F., Jeffries J.M.C., Svergun D. (2017). ATSAS 2.8: a comprehensive data analysis suite for small-angle scattering from macromolecular solutions. J. Appl. Cryst..

[bib8] Franke D., Jeffries C.M., Svergun D.I. (2018). Machine learning methods for X-ray scattering data analysis from biomacromolecular solutions. Biophys. J..

[bib9] Freire D.M.G.C., Garza-Garcia A., Grabowska A.D., Sala A.J., Ariyachaokun K., Panikova T., Beckham K., Pogenberg C.A.V., Cianci M.T.A. (2019). An NAD+ phosphorylase toxin triggers Mycobacterium tuberculosis cell death. Mol. Cell.

[bib10] Grant T. (2018). Ab initio electron density determination directly from solution scattering data. Nat. Methods.

[bib11] Hajizadeh N.R., Franke D., Jeffries C.M., Svergun D.I. (2018). Consensus Bayesian assessment of protein molecular mass from solution X-ray scattering data. Sci. Rep..

[bib12] Hirata A., Okada K., Yoshii K., Shiraishi H., Saijo S., Yonezawa K., Shimizu N., Hori H. (2019). Structure of tRNA methyltransferase complex of Trm7 and Trm734 reveals a novel binding interface for tRNA recognition. Nucleic Acids Res..

[bib13] Hura G.L., Budworth H., Dyer K.N., Rambo R.P., Hammel M., McMurray C.T., Tainer J.A. (2013). Comprehensive macromolecular conformations mapped by quantitative SAXS analyses. Nat. Methods.

[bib14] Konarev P., Svergun D. (2015). A posteriori determination of the useful data range for small-angle scattering experiments on dilute monodisperse systems. IUCr J..

[bib15] Luo M., Christgen S., Sanyal N., Arentson B.W., Becker D.F., Tanner J.J. (2014). Evidence that the C-terminal domain of a type B PutA protein contributes to aldehyde dehydrogenase activity and substrate channeling. Biochemistry.

[bib16] Manalastas-Cantos K., Kschonsak M., Haering C.H., Svergun D.I. (2019). Solution structure and flexibility of the condensin HEAT-repeat subunit Ycg1. J. Biol. Chem..

[bib17] Molodenskiy D.S., Mertens H.D., Svergun D.I. (2020). An automated data processing and analysis pipeline for transmembrane proteins in detergent solutions. Sci. Rep..

[bib18] Panjkovich A., Svergun D.I. (2015). Deciphering conformational transitions of proteins by small angle X-ray scattering and normal mode analysis. Phys. Chem. Chem. Phys..

[bib19] Petoukhov M., Franke D., Shkumatov A., Tria G., Kikhney A., Gajda M., Gorba C., Mertens H., Konarev P., Svergun D. (2012). New developments in the ATSAS program package for small-angle scattering data analysis. J. Appl. Cryst..

[bib20] Pettersen E.F., Goddard T.D., Huang C.C., Couch G.S., Greenblatt D.M., Meng E.C., Ferrin T.E. (2004). UCSF Chimera--a visualization system for exploratory research and analysis. J. Comput. Chem..

[bib21] Rambo R.P., Tainer J.A. (2013). Accurate assessment of mass, models and resolution by small-angle scattering. Nature.

[bib22] Różycki B., Kim Y., Hummer G. (2011). SAXS ensemble refinement of ESCRT-III CHMP3 conformational transitions. Structure.

[bib23] Schneidman-Duhovny D., Hammel M., Tainer J.A., Sali A. (2016). FoXS, FoXSDock and MultiFoXS: single-state and multi-state structural modeling of proteins and their complexes based on SAXS profiles. Nucleic Acids Res..

[bib24] Svergun D., Barberato C., Koch M. (1995). CRYSOL - a program to evaluate X-ray solution scattering of biological macromolecules from atomic coordinates. J. Appl. Cryst..

[bib25] Svergun D.I., Koch M.H., Timmins P.A., May R.P. (2013).

[bib26] Tarini M., Cignoni P., Montani C. (2006). Ambient occlusion and edge cueing for enhancing real time molecular visualization. IEEE Trans. Visual. Comput. Graph..

[bib27] Volkov V.V., Svergun D.I. (2003). Uniqueness of ab initio shape determination in small-angle scattering. J. Appl. Crystallogr..

[bib28] Watson M.C., Curtis J.E. (2014). Probing the average local structure of biomolecules using small-angle scattering and scaling laws. Biophys. J..

[bib29] Wright G.S., Watanabe T.F., Amporndanai K., Plotkin S.S., Cashman N.R., Antonyuk S.V., Hasnain S.S. (2020). Purification and structural characterization of aggregation-prone human TDP-43 involved in neurodegenerative diseases. iScience.

[bib30] Xu A.Y., Castellanos M.M., Mattison K.K.S., Curtis J.E. (2019). Studying excipient modulated physical stability and viscosity of monoclonal antibody formulations using small-angle scattering. Mol. Pharm..

